# Systematic review into factors associated with the recruitment crisis in psychiatry in the UK: students', trainees' and consultants' views

**DOI:** 10.1192/pb.bp.116.055269

**Published:** 2017-12

**Authors:** Abid Choudry, Saeed Farooq

**Affiliations:** 1 Leicestershire Partnership NHS Trust; 2 Keele University; 3 South Staffordshire and Shropshire NHS Foundation Trust

## Abstract

**Aims and method** To review the literature to examine the factors that may be affecting recruitment into psychiatry in the UK. We systematically searched four databases to identify studies from 1974 to 2016 and identified 27 papers that met the specified inclusion criteria.

**Results** Most papers (*n* = 24) were based on questionnaire surveys. The population in all studies comprised of 1879 psychiatrists, 6733 students and 220 746 trainees. About 4–7% of students opt for a career in psychiatry. Enrichment activities helped to attract students more towards psychiatry than just total time spent in the specialty. Job content in terms of the lack of scientific basis, poor prognosis and stigma towards psychiatry, work-related stress and problems with training jobs were common barriers highlighted among students and trainees, affecting recruitment. Job satisfaction and family-friendly status of psychiatry was rated highly by students, with lifestyle factors appearing to be important for trainees who tend to choose psychiatry.

**Clinical implications** Negative attitudes and stigma towards psychiatry continue to persist. Teaching and training in psychiatry needs rethinking to improve student experience and recruitment into the specialty.

Psychiatry as a career has been negatively regarded and unpopular among medical students. About 3.6% of British graduates decide on a career in psychiatry whereas about 6% are needed.^1^ From 2000 to 2011, the absolute number of consultant psychiatrists in England rose from 2904 to 4394 (an increase of 51%), with similar increases in Scotland and Wales, in line with the expansion of the consultant workforce in most secondary care specialties.^1^ A need for more psychiatric consultant posts has been predicted based on projections of increasing workloads due to the increased population needs.^2^ A perceived fall in the proportion of UK medical school graduates choosing a postgraduate career in psychiatry, and low competition ratios for first-year core specialist training (CT1) posts in psychiatry,^3–5^ has led to many questions about the way in which psychiatry is taught at medical school and how psychiatry could be made as an attractive career option.^6–8^

Low recruitment in psychiatry is a global issue, highlighted in a number of publications.^9,10^ However, the problem also has country-specific dimensions, as each country has different factors such as the pay scale for the specialty, medical education system and training programmes, which may attract or dissuade medical students from a career in psychiatry. The subject has not been reviewed systematically especially in the context of recruitment in the UK. The review question was: what are the factors that influence recruitment to psychiatry as a career choice and how can this be improved? The aim of this paper is to systematically review the literature from the UK on psychiatry as a career choice. We are specifically interested in identifying the factors that influence the recruitment into psychiatry with emphasis on barriers and facilitators affecting recruitment. A greater understanding of the views ranging from medical students to trainees and consultants working in the UK may enable curriculum developers and recruitment leads to design programmes that attract students and trainees to psychiatry as a career and improve students' attitudes towards psychiatry in the long term.

## Method

We followed the PRISMA statement^11^ as a guide for conducting the systematic review. The following electronic databases were searched from 1974 onwards: Medline, EMBASE, CINAHL, PsycINFO. These databases were selected in view of the fact that almost all the literature published from the UK would be covered in these databases. Keywords used in the searches included: medical students, trainees, doctors, consultant, attitudes, psychiatry, career, undergraduate psychiatry. Boolean operations and truncations were added to allow for alternative endings in the keyword searches. The search was last updated in August 2016.

We included studies that described a UK sample, were published in English in a peer-reviewed journal and provided original data on careers in psychiatry or factors affecting recruitment in psychiatry. We excluded studies that had the primary objective of addressing other issues that were not directly relevant to recruitment in psychiatry but only mentioned their effects on career choice as a secondary effect. Studies which provided data on medical careers in general were included if these provided data relevant to psychiatric careers or recruitment.

**Fig. 1:**
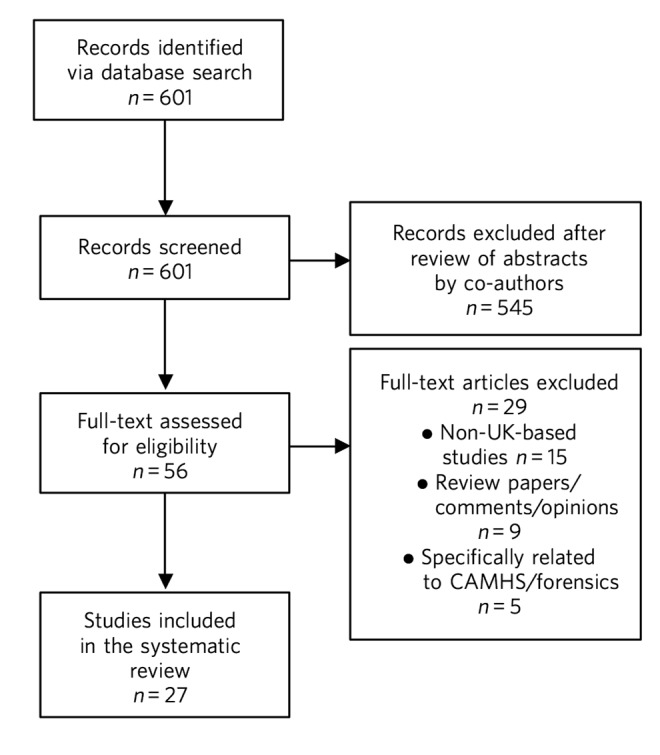
Summary of the abstracts reviewed to identify relevant papers. CAMHS, child and adolescent mental health services.

The electronic search returned 601 relevant abstracts and titles. We screened the titles and abstracts and excluded the studies that were not directly relevant to the objectives of the review. Therefore we excluded the studies that did not describe a UK sample or did not provide the original data. We also excluded the studies that described the recruitment to certain subspecialties and therefore not relevant to the recruitment to psychiatry in general. After screening these titles and abstracts we decided to examine 56 full text papers. Finally, we included 27 papers in the review. Further details are provided in Fig. 1. The studies were conducted in a number of different populations, settings and periods, and also used diverse methodologies varying from surveys to statistics derived from databases. The 30-item Attitudes Towards Psychiatry (ATP-30),^12^ a validated tool used to assess attitudes of students towards psychiatry, was used in four papers; other papers used different questionnaires to assess attitudes. Due to the diverse methodologies it was inappropriate to pool the data to produce a statistical summary. We therefore describe the main findings and produce a narrative summary of results.

## Results

### Characteristics of included studies

Table 1 shows the characteristics of the 27 studies included in the review. The majority of studies (*n* = 24) were surveys and questionnaire-based studies. The other studies included one database study of doctors entering training posts and two retrospective cohort studies, which looked at which medical school psychiatrists had attended. These reported on medical students (*n* = 12), trainees (*n* = 10), medical students and trainees (*n* = 1), trainees and consultants (*n* = 1) and consultants (*n* = 3). One of these studies looked at data from postgraduate departments in different medical schools, and one study reported on the database of applicants to MMC (Modernising Medical Careers) for training posts.

**Table 1:** Study characteristics

Author/year	Population	Method/design	Total sample, *n*	M:F ratio, %/ response rate, %
Brook, 1976^19^	Medical school of origin for psychiatrists	Retrospective cohort	531	No data provided

Brook, 1983^21^	Medical school of origin for psychiatrists	Retrospective cohort	1229	No data provided

Levine *et al*, 1983^24^	Medical students	Survey	30	No M:F data/100

Brook *et al*, 1986^20^	Medical students	Survey	498	64:36/40

Calvert *et al*, 1999^33^	Medical students	Survey	392	M:F, no significant difference/70

Mcparland *et al*, 2003^30^	Medical students	Cohort study – survey	379	54:46/84

Maidment *et al*, 2003^31^	Medical students	Survey	837	33.6:66.4/99.6

Petrides & McManus, 2004^23^	Medical students	Cohort study – survey	8283	Only descriptive data provided in this paper

Rajagopal *et al*, 2004^35^	Medical students	Survey	301	31.9:68.1/52

Curtis-Barton & Eagles, 2011^25^	Medical students	Survey	467	44:64/51

Budd *et al*, 2011^26^	Medical students at 4 different medical schools	Survey	905	36:64/Not provided clearly reported range 50–85% depending on medical school

Archdall *et al*, 2013^34^	Medical students	Survey	15	30:70/100

Halder *et al*, 2013^28^	Medical students – 18 UK medical schools	Survey	484	34:66/None

Farooq *et al*, 2014^27^	Medical students (only UK data used)	Survey	291	Males 37:63/16

Maidment *et al*, 2004^31^	Trainee doctors	Survey	234	52.1:47.9/67.4

Goldacre *et al*, 2005^14^	Trainee doctors (graduates from 1974 to 2000, UK medical schools)	Survey	21 845 year 1 17 741 year 3	Year 1 55:45/75 Year 3 56:44/74

Lambert *et al*, 2006^15^	Trainee doctors	Survey	572	No M:F data/74

Fazel *et al*, 2009^13^	Trainee doctors (all applicants to MMC for training posts)	Database analysis	31 434	No M:F data

Barras & Harris, 2012^38^	Trainee doctors	Survey	359	52.2:47.8/16.1

Goldacre *et al*, 2012^16^	Trainee doctors who had qualified in 2002, 2005, 2008	Survey	9155	37:63/56

Goldacre *et al*, 2013^17^	Trainee doctors who qualified between 1974 and 2009	Survey	33 974	Year 1 49:51/65.9 Year 3 51:49/68.6 Year 5 52:48/69.5

Svirko *et al*, 2013^18^	Trainee doctors who qualified 2005, 2008, 2009	Survey	9348	46.9:55.8/52.2

Collier & Moreton, 2013^22^	Hospital postgraduate departments of 19 medical schools	Survey	19 medical schools	No M:F data/70

Woolf *et al*, 2015^37^	Medical students and trainee doctors	Cohort study – survey	105	34:66/50

Korszun *et al*, 2011^36^	Trainee doctors, academics, trust clinicians	Survey	309	61.5:38.5/None

Dein *et al*, 2007^39^	Consultants	Survey	72	68:32/82.7

Denman *et al*, 2016^32^	Consultants and trainees	Survey	Consultants: 47 Trainees: 51	55:45/41.7 (consultants 42%, trainees 41%)

M, male; F, female; MMC, Modernising Medical Careers.

a. Data not used from this paper just broad findings in review.

In survey-based studies response rates varied from 16% to 100%. Mean response rate from the papers which had figures available (*n* = 20) was 63.3%. The population in all studies comprised of 1879 psychiatrists, 6733 students and 220 746 trainees. One database study^13^ looked at 31 434 trainee doctors, and studies by Goldacre *et al* were aimed at all doctors in training, accounting for large numbers of respondents in the trainee subcategory.^14–18^

### The influence of the medical school and teaching practices

Most of the research addressing the influence of the medical school and teaching practices on selecting psychiatry as a career was carried out in the 1970's and 1980's. Two studies by Brook *et al*^19,20^ looked at the medical school of origin for 531 psychiatrists between 1961 and 1970 and reported no significant relationship between schools that had a professional unit or specific teaching programme and students pursuing psychiatry in the long term. However, it was noted that those schools that produced fewer psychiatrists tended to have either a recently established professional unit or none.^19^

No clear pattern emerged in terms of the type of teaching offered at each university and the impact this had on choice of psychiatry as a career in the long term.^19^ All four Scottish schools, and Cambridge and Oxford were noted to be higher in terms of producing psychiatrists, attributed possibly to the well-established professional units such as the Maudsley and Bethlem Royal hospitals.^19^ The personality, charisma and enthusiasm of teachers were associated with an increase in the uptake of psychiatry in the long term.^19,21^

Brook *et al*^21^ found that the effectiveness of teaching rather that the amount of teaching had an effect on student attitudes and recruitment into psychiatry The attitude of non-psychiatric teachers appeared to be influential with doctors experiencing negative attitudes of other doctors towards psychiatry.^21^ The two hospitals which ranked top in terms of producing psychiatrists had changed their teaching model. One stressed the importance of psychiatry as being part of general medicine, emphasising the effectiveness of physical therapy, whereas the other placed emphasis on liaison psychiatry and psychotherapy.^21^

More recent work by Collier *et al*^22^ looked into the teaching time allocated for psychiatry in foundation programmes across the country. They found that only 2.3% of teaching was dedicated to psychiatry compared with 44.1% to medical and surgical topics.^22^ Exposure to psychiatry remained limited with 4 out of 17 hospitals in the survey not having any teaching on psychiatry for medical students.^22^ Doctors generally led a higher proportion of medicine and surgery teaching sessions (63%) compared with psychiatry (48%).^22^

### Medical students' views of psychiatry and factors affecting career choice

Twelve studies examined the factors affecting medical students' career choice and one study looked at both medical students and trainees. Petrides *et al*^23^ studied the theoretical understanding of how different medical specialties are perceived and how choices are made. Psychiatrists were found to have a more artistic approach to medicine, seeing interpreting and responding imaginatively to a range of medical, social, ethical and other problems. This is in keeping with early work by Levine *et al*^24^ who also found that there was a group of students who were ‘psychologically minded’ and they could be identified and encouraged to make psychiatry as career choice.

Budd *et al*^26^ found that job satisfaction (98%, *n* = 128) and family-friendly status of psychiatry (79%, *n* = 103) were important for students who rated psychiatry as one of their top three choices.^26^ The academic status was significantly less important (48%) for students who placed psychiatry as their top three specialty schools *v.* 63% for those who did not place psychiatry in their top three choice.^26^

### Choice of psychiatry as a career among medical and sixth form students

The number of students choosing psychiatry has remained fairly stable at around 4–7%.^25–27^ Three per cent of students from six medical schools placed psychiatry as their first choice, with 18% seriously considering it.^20^ Halder *et al*^28^ found similar results in 18 medical schools; 16% chose psychiatry as a future career on entering medical school but by the final year only 3% had decided to pursue a career in the subject. These results were replicated by Farooq *et al*^27^ In a survey of sixth form students, Maidment *et al*^29^ reported that 60.9% (*n* = 363) indicated that it would be very likely or they would definitely want to pursue psychiatry as a career. In terms of overall intentions to pursue a career in a specialty, the ratings for psychiatry was similar to general medicine at 12.4% (*n* = 72) *v.* 12.2% (*n* = 69) respectively.^29^

### Effect of undergraduate attachment and education on choosing psychiatry as a career

Positive attitudes towards psychiatry and the influence by a teacher during the attachment correlated with an intention to purse psychiatry as a career in the long term.^29–31^ Three studies highlighted the importance of psychiatric attachment. Student attitudes improved as the attachment progressed.^26,30,31^ Maidment *et al*^29^ found 1.4% of fourth-year medical students expressed a definite intention to pursue which rose to 4.7% after their attachment.^29^ McParland *et al*^30^ reported that 19% (*n* = 58/309) of students were very attracted to psychiatry or had a definite intention to pursue psychiatry at the start of the placement, which increased to 27% (*n* = 101/373) of students at the end of the attachment. The importance of the undergraduate experience was highlighted by a recent study showing 50% of consultants and 37% of trainees surveyed decided on a career in psychiatry while still at medical school.^32^

Calvert *et al*^33^ looked into the attitudes of medical students towards psychiatry and psychiatric patients at year 1, 3 and 5 in medical school. First-year medical students were more likely to have stereotypical views compared with third- and fifth-year students, and were more likely to agree with statements such as ‘Psychiatry deals with imaginary illness’ (mean 1.4, s.d. = 0.9, *P* < 0.5).^33^ Fifth-year students (mean 3.2, s.d. = 1.4) showed lower agreement than third-year medical students (mean 3.6, s.d. = 1.2, *P* < 0.5) with the statement ‘Psychiatry is as a challenging career’.^33^ As they progressed through medical school, students recognised that mental illness has serious morbidity and that people do recover from mental illness,^33^ showing that attitudes towards psychiatric patients improved with greater clinical experience but possibly became more negative towards psychiatry as a career.

Other factors that appeared to affect students positively included enrichment activities, i.e. activities beyond standard teaching and clinical placements led to a significantly increased interest in psychiatry.^28^ These included research experience in psychiatry (13% *v.* 4% in those not interested in psychiatry, *P* = 0.001), university psychiatry clubs (38% *v.* 11%, *P* < 0.001), psychiatry electives (14% *v.* 1%, *P* < 0.001) and psychiatry special study modules (38% *v.* 16%, *P* < 0.001).^28^

McParland *et al*^30^ identified factors which increased interest in psychiatry, including: influence or encouragement by someone during the attachment (74%, *n* = 282), particularly the influence by consultants (43%, *n* = 163), exposure to interesting and stimulating ideas (29%, *n* = 110), liking someone's approach (27%, *n* = 103), feeling someone believed in their ability (11%, *n* = 41) and having formed close working relationships (9%, *n* = 33).^30^ Other factors that had a significant impact were: receiving encouragement from the consultants (*n* = 374, *P* < 0.001, *r* = 0.26), seeing patients respond to treatment (*n* = 374, *P* < 0.001, *r* = 0.20) and having a direct role in the involvement of patient care (*n* = 374, *P* < 0.001, *r* = 0.26).^30^

### Factors which did not affect career choice of medical students

Seeing patients in different settings or different phases of the illness had no effect on career choice of medical students when deciding their career intentions.^28^ Interestingly, one study found that the earning potential and status of psychiatry had no effect on selecting psychiatry as a career choice.^26^ Other factors related to teaching such as quality of rating of small group teaching and lectures,^28^ the curriculum type used^30,31^ and performance at viva examinations and multiple choice questions also had no effect on the career choice.^31^

### Factors discouraging medical students to choose psychiatry as a career

Curtis-Barton *et al*^25^ in their survey (*n* = 467) found that the factors discouraging students to pursue a career in psychiatry included: prognosis of patients (62%), perception that there is a lack of evidence in diagnosis (51%), lack of scientific basis (53%) and the amount of bureaucracy and paperwork in the specialty (48%). Other discouraging factors included the stigma towards psychiatry (30%), the standing of the profession among medical colleagues (31%) and comments by other specialists (26%).^25,34^ Psychiatry scored the lowest among the specialties as a career choice. Students described psychiatry as boring, unscientific, depressing, stressful, frustrating and ‘not enjoying the rotation’.^35^

Many students experienced psychiatry as being different to other specialties. For some this was a reason not to pursue psychiatry as a career but for others it was a positive aspect of the specialty Students felt ward rounds focused on ‘social issues’ rather than medical conditions. Some found it an ‘emotional burden’ and others felt that psychiatry could not ‘fix’ people and no one is being cured.^34^

A survey by Korszun *et al*^36^ examined the views of trainee, academics and clinicians on students not taking up psychiatry. The following factors were identified as deterring the students from psychiatry: negative attitudes towards psychiatrists from other doctors and health professionals (57%), stigmatisation of psychiatry (40%), stigma associated with mental health disorders (39%), poor teaching and role modelling from psychiatrists (37%), psychiatry not seen as medical or scientific enough (26%) and poor morale among psychiatrists (26%).^36^

### Factors affecting trainees' and consultants' choice of psychiatry

Fazel *et al*^13^ found that psychiatry was the sixth most popular specialty out of ten specialty groups for trainees applying for training places. A higher proportion of female graduates were shown to choose psychiatry between 1974 (32%) and 1999 (59%).^13^ However, a more recent survey showed a slight decline in the number of women choosing psychiatry over the last decade, 4.9% (1999) *v.* 4.6 % (2009).^17^

Goldacre *et al*^14^ examined career choices for medical students over the past 40 years. The number of doctors choosing psychiatry as a career has hardly changed and remains around 4–5%, which is similar to figures from 1975.^14^ It was noted that students who went on to work in psychiatry 10 years after graduation, 52% (224 out of 428) had chosen psychiatry in the first year after graduation and 71% (308 out of 434) had chosen it in year 3.^14^

In common with the factors attracting students towards psychiatry, numerous studies identified factors that appear to attract trainees towards psychiatry The major attractions for choosing psychiatry are listed in Box 1. Denman *et al*^32^ found that the most common factor influencing core trainees' (60%) and consultants' (70%) decisions to specialise in psychiatry was emphasis on the patient as a whole.^32^ Trainees highlighted that mental health was an area of need (53%) and empathy and concerns for people with mental illness (53%) were important reasons for choosing psychiatry.^32^

**Box 1** Factors attracting medical students and trainees in pursuing psychiatry as a career

**Medical students**

- Encouragement by colleagues^24,26,30,31^
- Influence by someone during the placement^24,30^
- Females are more likely to favour a career in psychiatry^24,27,30^
- Family history of mental illness was associated with choosing psychiatry^24^
- Quality of experience^26,27,30,33^
- Role models can have a positive impact on students pursuing a career in psychiatry^28,30,33^
- Enrichment activities^27,28^

**Trainees**

- Hours and conditions of work^17,31,32^
- The doctor's personal assessment of their aptitudes and skills,^17,31,32^ for example recognising factors such as using one's intellect to help others^37^
- Experience of the subject as a student^17,31^
- Inclinations before medical school and a positive student experience^17,37^
- Attitudes and inclination to psychiatry as a medical student^24,31,37^
- Lifestyle factors^32,37^
- Encouragement from consultants and senior doctors^31^
- Emphasis on the patient as a whole person and empathy/concern for mentally ill people^32^

### Barriers associated with not choosing psychiatry as a long-term career choice for trainees

Barras & Harris^38^ explored trainee's experiences (*n* = 359) within psychiatry. Trainee attitudes were grouped into different categories. The attitudes towards psychiatry (12.6%), professional role (12%) and day-to-day working (11.3%) were identified as the main negative factors. Trainees raised concerns with the training programmes in psychiatry, such as problems with the rota and not having enough time with patients.^38^ Many trainees felt frustrated with the Annual Review of Competence Progression (ARCP) process and workplace-based assessments, as well as the duplication of paperwork being a constant frustration.^38^

The studies identified a number of barriers against choosing psychiatry as a career (Box 2).

Trainees felt improvements were needed in terms of training opportunities and felt this could be enhanced by providing a variety of jobs, increasing research opportunities and increasing medical aspects of training.^38^

Work looking into consultant psychiatrists' views into why they chose psychiatry was limited to two papers.^32,39^ Dein *et al*^39^ found that the majority of consultants (46%) chose psychiatry as a career soon after leaving medical school, and a recent study surveying consultants in the West Midlands found that 50% had made their choice by graduation from medical school.^32^ The main reasons cited by consultants for choosing psychiatry as a career included: empathy for those with a mental disorder (36.1%), interface with neuroscience (25%), expectation of better working conditions in psychiatry (20%) and influence of teaching at medical school (19.4%).^39^ Denman *et al*^32^ highlighted several ‘very important’ reasons for consultants choosing psychiatry including: career in psychiatry would be intellectually challenging (60%), sense of fulfilment expected from seeing patients improve (47%) and enjoyment of problem-solving (47%). Lifestyle factors such as salary, better working conditions and quality of life were shown to be more important reasons for choosing psychiatry for trainees compared with consultants.^32^

**Box 2** Barriers associated with not choosing psychiatry as a career

- Job content (71.7%) (*n* = 71) (including the lack of scientific basis, job not being clinical, poor prognosis)^15,17,25,35,36,38^
- Poor public image of psychiatry^15,25,36^
- Lack of respect towards psychiatry as a specialty by other specialties^15,25,36^
- Work-related stress cited by (49%)^15^ trainees in psychiatry^38^
- 25–50% of trainees leaving psychiatry as a specialty cited lack of resources as one of the main reasons which was significantly more than those rejecting general practice and trauma and orthopaedics^15,38^
- 25–50% of trainees leaving psychiatry^15,16,38^ highlighted:
  - lack of adequately supervised training
  - lack of evidence base to diagnosis and treatment
  - lack of improvement in patients
  - work-life balance
  - work not clinical enough
- Physical risks involved in the job^15^
- Sense of eroded professionalism^36,38^
- Too much paperwork and duplication^25,36,38^
- Problems with rota and not enough time with patients^38^
- Trainees leaving the scheme felt frustrated with workplace-based assessments^38^
- Low morale among workforce^33^
- Future role of psychiatrists being eroded^33^

## Discussion

This is the first systematic review of literature which examined factors that influence the choice of psychiatry as a career in the UK. The main findings are that enrichment activities help to attract students more towards psychiatry than just total time spent in the specialty. Job satisfaction and family-friendly status of psychiatry was rated highly by students who tend to choose psychiatry. Role models and encouragement from consultants may increase the number of students who want to pursue psychiatry as a career. The major factors that appeared to dissuade medical students/trainees from pursuing psychiatry as a career included: an apparent lack of scientific basis of psychiatry and work not being clinical enough, perception that psychiatry is more concerned about social issues, the bureaucracy, paperwork, apparent poor prognosis of patients, stigma towards psychiatry as a specialty, low morale, and onerous workloads as a consultant.

We are aware of one previous systematic review that examined medical students' attitudes towards psychiatry internationally.^40^ In common with our study this systematic review alongside another survey of psychiatrists^36^ highlighted the stigma towards mental illness as a major barrier influencing negative medical views towards psychiatry.^40^ Stigma towards psychiatry as a specialty arises from a variety of sources, notably from medical students themselves. In addition, this stigma could arise from ward staff attitudes towards patients and from other doctors in other specialties, which detract students.^13,25,34^

Other reviews have looked at one aspect such as the effect of clinical experience of psychiatry on medical students' attitudes towards the specialty.^41^ Lyons^40^ highlighted the impact of poor-quality teaching leading to negative attitudes towards psychiatry and highlighted the need to address psychiatry curricula and introduce novel teaching strategies.^40^ El-Sayeh *et al*^42^ have previously highlighted the importance of teaching and the various methods which could be utilised to try to improve the student experience and in turn help attract students towards psychiatry. The recent survey by Korszun *et al*^36^ highlighted that the number of clinicians compared with academics and trainees agreed that they did not have time to teach medical students (*P* < 0.001). Both clinicians (42%) and academics (47%) felt that teaching medical students did not contribute to their future career prospects compared with 21% of trainees (*P* < 0.001)^36^ Fewer clinicians considered teaching to be a significant component of their appraisal compared with trainees and academics.^36^ The combination of poor teaching practices due to lack of resources or commitment and the stigma reinforce the poor image of psychiatry.

We feel that the findings of our study support the recommendations made by Mukherjee *et al*^43^ which identify a number of steps to address the crisis in psychiatry recruitment at different nodal points in a medical career, i.e. prior to entry to medical school, during medical education and after graduation.

### Improving recruitment

This study highlights the need to change the experience of psychiatry at undergraduate and postgraduate level in keeping with work by Shah *et al*^44^ who highlighted early medical experience, influence of seniors and the aspects related to working environment as areas that could be affected positively which in turn could have a positive effect on choosing psychiatry as a career. Kelley *et al*^45^ highlighted the impact of foundation programme experience in psychiatry, with a significantly higher proportion of trainees pursuing a career in psychiatry compared with those without any exposure to psychiatry (14.9% *v.* 1.8%). This correlates with earlier findings by Shah *et al*^44^ that found a significant correlation between those Scottish students considering psychiatry as a career and having held a psychiatry post.^43^ A recent survey by Denman *et al*^32^ showed that 43% of psychiatry trainees made their decision to specialise in psychiatry during the foundation years, correlating with increased exposure to psychiatry during the foundation years with 80% of trainees in this survey completing a post in psychiatry during the foundation years. Specific enrichment activities beyond standard teaching and clinical placements such as research experience in psychiatry, university psychiatry clubs, summer schools,^46^ psychiatry electives and psychiatry special study modules appear to be a way in which medical students will gain invaluable experiences and improve their attitudes to psychiatry. These need to be adopted and evaluated in future programmes to enhance recruitment in psychiatry Collier *et al*^22^ found that only 2.3% of teaching was dedicated to psychiatry compared with 44.1% to medical and surgical topics, which does not help the poor image of psychiatry. This and similar issues need to be addressed at institutional level.

A number of studies found that psychiatry has a perception that it is not a ‘scientific’ or ‘medical’ discipline.^15,36,38^ Medical students and trainees expressed the views about the weakened medical identity of psychiatry. The erosion of the role of the psychiatrist was cited by some psychiatry trainees as a potential factor that would make them consider leaving psychiatry training.^38^ This unfortunately is not helped by negative comments or ‘bad-mouthing’ of psychiatry.^36,47^

Interventions such as anti-stigma films and Medfest^48^ have been shown to improve medical students' attitudes to psychiatrists, serious mental illness and psychiatry, at least in the short term.^48,49^ However, it appears that there is need to reconsider the content of psychiatric training and the undergraduate curriculum. It has been suggested that moving undergraduate teaching from in-patient to general hospital settings such as liaison psychiatry will allow students to see patients with problems that are relevant to medical practice.^47^ Setting up and evaluating such programmes that have the potential to offer a different and enjoyable experience for medical students and foundation doctors should be a priority to improve the image of and recruitment into psychiatry.

### Limitations

A limitation of the study is that almost all data are based on surveys and databases. This represents a cross-sectional view on the subject. The lack of any comparisons with other specialties, which may have similar recruitment rates, is particularly concerning. The focus on UK studies is also a limitation but was necessary to understand the factors affecting recruitment in this country We noted with some concern that there are only a few studies that address the positive aspects of psychiatry,^26–28,30–33^ which may attract students and trainees towards psychiatry, and how these can be used for improving the recruitment. Future studies need to address this gap in the literature. Finally, we feel that the problems underlying the recruitment in psychiatry perhaps also reflect the lack of parity of esteem. Unless mental health is valued equally with physical health, the misconceptions and distorted perceptions about psychiatry as a discipline in which a medical career can be fruitfully pursued will linger on and will hinder aspiring physicians from considering psychiatry as a career option.
